# Behavior of human gastrocnemius muscle fascicles during ramped submaximal isometric contractions

**DOI:** 10.14814/phy2.12947

**Published:** 2016-09-07

**Authors:** Martin E. Héroux, Peter W. Stubbs, Robert D. Herbert

**Affiliations:** ^1^ Neuroscience Research Australia Randwick New South Wales Australia; ^2^ School of Medical Sciences University of New South Wales Randwick New South Wales Australia

**Keywords:** Fascicle length, fascicle pennation angle, isometric contraction, muscle

## Abstract

Precise estimates of muscle architecture are necessary to understand and model muscle mechanics. The primary aim of this study was to estimate continuous changes in fascicle length and pennation angle in human gastrocnemius muscles during ramped plantar flexor contractions at two ankle angles. The secondary aim was to determine whether these changes differ between proximal and distal fascicles. Fifteen healthy subjects performed ramped contractions (0–25% MVC) as ultrasound images were recorded from the medial (MG, eight sites) and lateral (LG, six sites) gastrocnemius muscle with the ankle at 90° and 120° (larger angles correspond to shorter muscle lengths). In all subjects, fascicles progressively shortened with increasing torque. MG fascicles shortened 5.8 mm (11.1%) at 90° and 4.5 mm (12.1%) at 120°, whereas LG muscle fascicles shortened 5.1 mm (8.8%) at both ankle angles. MG pennation angle increased 1.4° at 90° and 4.9° at 120°, and LG pennation angle decreased 0.3° at 90° and increased 2.6° at 120°. Muscle architecture changes were similar in proximal and distal fascicles at both ankle angles. This is the first study to describe continuous changes in fascicle length and pennation angle in the human gastrocnemius muscle during ramped isometric contractions. Very similar changes occurred in proximal and distal muscle regions. These findings are relevant to studies modeling active muscle mechanics.

## Introduction

The human gastrocnemius is a bipennate muscle with fascicles that extend between superficial and deep aponeuroses. When the knee and ankle are not permitted to move and the gastrocnemius muscle contracts maximally, fascicles shorten by 28–42% and their angle of pennation increases by 80–250% (Narici et al. 1996; Kawakami et al. 1998; Maganaris et al. 1998; Martin et al. 2001). The magnitude of these changes depends on whether fascicles are located in the medial or lateral part of the muscle and the angle of the ankle when the contraction occurs (Kawakami et al. 1998; Maganaris et al. 1998).

Comparatively, little is known about how human gastrocnemius muscle architecture changes during submaximal contractions, especially weak contractions. Weak contractions are particularly relevant because the majority of daily activities require only a fraction of the force generation capabilities of our muscles (Kern et al. 2001). Studies of brief static contractions performed in increasing steps of 10–20% of maximal torque have concluded that muscle fascicle length decreases and pennation angle increases either linearly or quadratically (Narici et al. 1996; Maganaris et al. 1998). These studies were small (six subjects), performed a single recording at each contraction intensity, and only included 2–3 data points at low‐to‐moderate contraction intensities.

Studies using surface electromyography recordings and MRI have reported the presence of localized activity in the gastrocnemius muscle (Kinugasa et al. 2005; Vieira et al. 2010, 2011; Hodson‐Tole et al. 2013; Csapo et al. 2015). Although there are conflicting results (e.g., Vieira et al. 2010, 2011), the general view is that muscle fibers associated with low‐threshold motor units are preferentially located in the distal portion of the medial gastrocnemius muscle (Kinugasa et al. 2005; Vieira et al. 2010, 2011; Hodson‐Tole et al. 2013; Csapo et al. 2015). This is at odds with recent results obtained using indwelling motor unit recordings where over 40% of recorded units spanned at least half the length of the muscle, many of which spanned the entire length (Héroux et al. 2015). Although it has been argued that these results do not disprove the existence of regional muscle activity in the medial gastrocnemius muscle (Vieira et al. 2016), strong evidence to the contrary remains elusive (Blouin et al. 2016). If present, regional muscle activity – with low‐threshold motor units concentrated distally – should result in earlier shortening in distal muscle fascicles during ramped contractions.

The primary aim of this study was to obtain precise estimates of the continuous change in muscle fascicle length and muscle fascicle pennation angle in the human gastrocnemius muscle during ramped, submaximal isometric plantar flexor contractions performed at two ankle angles. The secondary aim of the study was to determine whether changes in muscle fascicle length and pennation angle differ between muscle fascicles in proximal and distal regions of the medial gastrocnemius muscle.

## Methods

Subjects were 15 healthy adults (mean age 29 years, range 23–46 years; seven female; mean mass 71 kg, range 54–95 kg; mean height 174 cm, range 160–198 cm; mean leg length 40.5 cm, range 36.0–45.0 cm) with no known musculoskeletal pathology on the tested leg. The procedures were approved by the University of New South Wales Human Research Ethics Committee and the study was conducted according to the Declaration of Helsinki. All subjects gave written, informed consent.

### Experimental protocol

**Figure 1:**
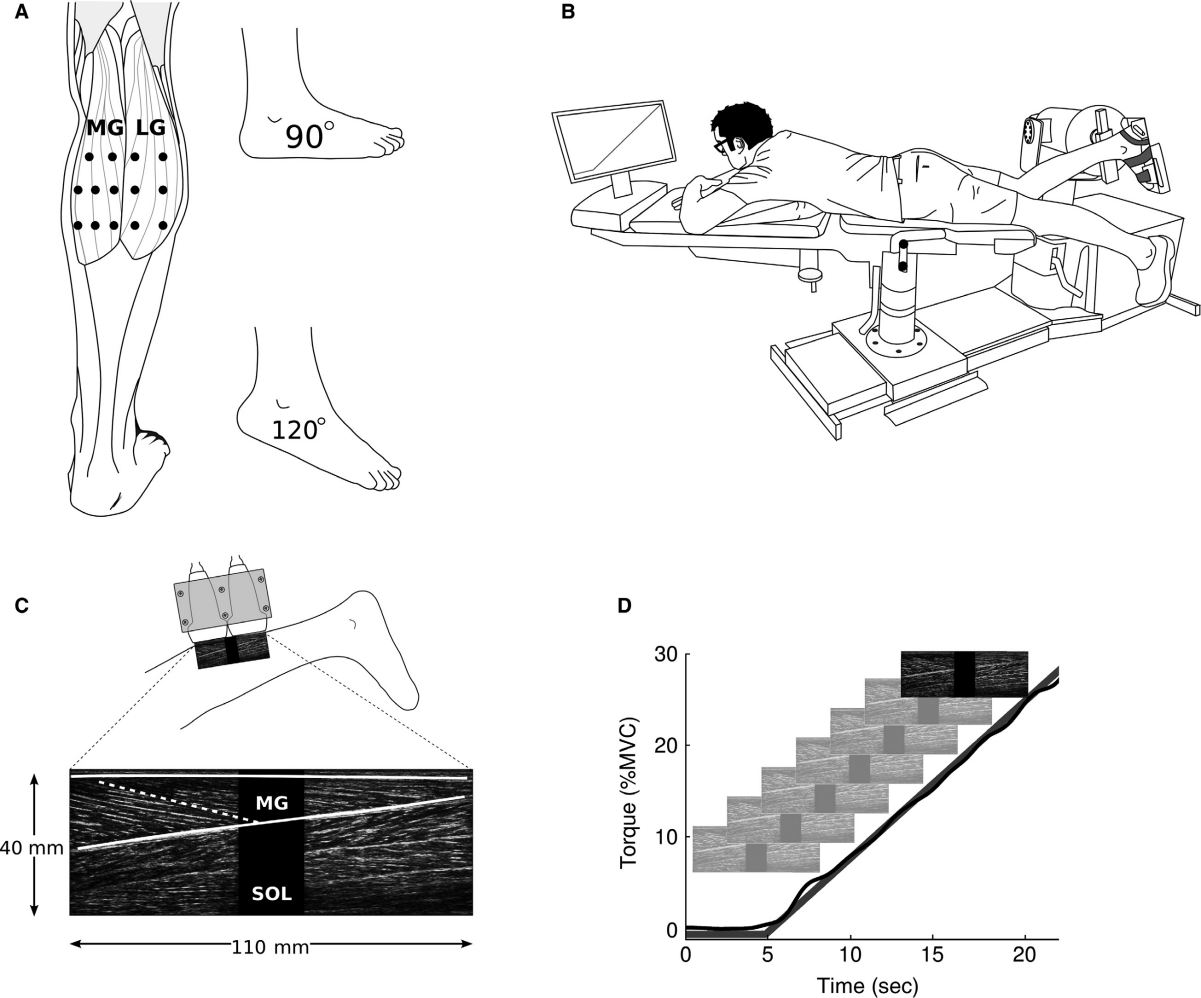
Experimental set‐up and data collection. (a) Ultrasound measures were taken at two different ankle angles from 14 target sites, eight located over the medial gastrocnemius (MG) and six over the lateral gastrocnemius (LG). (B) Subjects lay prone with the right ankle positioned in an isokinetic dynamometer. Visual feedback of torque produced during maximal and ramped isometric contractions was provided on a monitor. (C) Two ultrasound transducers in a custom‐built mold provided a composite image of 40 mm by 110 mm from which a target fascicle and superficial and deep aponeuroses were identified. (D) In each trial, subjects performed an isometric ramped contraction by tracking a visual target provided on the monitor. Ultrasound images were recorded at 10 Hz during these contractions.

With the subject standing, the margins of the right medial gastrocnemius (MG) and lateral gastrocnemius (LG) were palpated. Within those margins, 14 target sites were marked on the skin: eight over MG and six over LG (Fig. 1A). Subjects then lay prone with the right knee flexed 23–42° and the right foot placed on the footplate of an isokinetic dynamometer and secured with Velcro straps (Fig. 1A; Cybex Norm with Humac, CSMi, Stoughton MA). The axis of the dynamometer was aligned as closely as possible with the axis of the ankle. Subjects performed three maximal voluntary isometric plantar flexor contractions (MVC) with the ankle at 90° and 120° (Fig. 1A). The order of MVC testing was randomized across subjects. Verbal encouragement was provided, as was real‐time visual feedback of plantar flexion torque on a computer monitor. Each maximal contraction lasted 3–5 sec, with a 2 min rest between contractions. The maximum torque generated was identified for each ankle position.

Next, subjects performed two series of 14 isometric ramped contractions. One series was carried out with the ankle at 90° and the other with the ankle at 120°; the order of testing was randomized across subjects. The ramped isometric contractions required subjects to linearly increase plantar flexion torque from 0% to 30% of MVC over a period of 15 sec. Only data from 0% to 25% MVC were retained for analysis as it was not possible to reliably track muscle fascicle behavior at higher contraction intensities. The dynamometer measured voluntary isometric plantar flexion torque; visual feedback of this torque and the desired ramped contraction was presented on a monitor (Fig. 1B and D). Subjects performed 1–2 practice ramped contractions at the start of each series and had a 2 min rest between each ramped contraction.

As subjects performed ramped contractions, two ultrasound transducers (Esaote MyLab25 with LA522E 46 mm linear array, 7.5–12 MHz operating at 12 MHz; Esaote, Genoa, Italy) were used to generate images of the gastrocnemius muscle (Fig. 1C and D). The transducers were held together by a custom‐built mold. The use of two ultrasound transducers provided a wider field of view than would otherwise be possible (Herbert et al. 2011). During each trial, the ultrasound transducers were manually positioned so that the middle of the proximal transducer was located over one of the 14 target sites. The order in which sites were tested was randomized. The transducers were orientated to obtain the clearest possible image of a fascicle which terminated superficially under the middle of the proximal transducer. It was assumed that the plane in which the clearest image was obtained approximated the plane in which the muscle fascicle lay (Kwah et al. 2013; Bolsterlee et al. 2015). In some trials, the muscle moved underneath the transducer and it was necessary for the operator to rotate the transducer, usually by only a few degrees, to maintain a high‐quality image. Such trials occurred equally across 90° or 120° trials and when recording from various muscle regions. Given that multiple trials were recorded for each ankle position, measurement noise caused by these small rotations likely had little to no impact on average fascicle behavior.

Images from the two transducers were captured synchronously with a dual‐channel video capture card at 10 Hz with Spike2 software and the S2video plug‐in (CED, Cambridge, UK). The torque signal was sampled at 50 Hz using the same system (12–bit DAQ, 1401, CED, Cambridge, UK).

### Data analysis

For each trial, the two ultrasound video sequences – one from each transducer – were stitched together to form a video sequence of composite images with a 110 mm field of view (Fig. 1C). There was an 18 mm gap in the middle of the image because the ultrasound images did not extend to the edge of the transducers (Herbert et al. 2011). In the first frame of the video sequence, the proximal and distal ends of the target fascicle were identified. Occasionally, it was possible to clearly visualize the proximal and distal ends of a fascicle in the ultrasound image of each transducer; in these instances, two fascicles were identified. The two‐dimensional image coordinates of proximal and distal fascicle points were tracked through the video sequence using a cross‐correlation method (Herbert et al. 2011). Data from video sequences with poor image quality were not used; poor image quality typically occurred in the second half of ramped contractions and rendered accurate tracking of fascicle points impossible. This excluded, for the MG, eight trials at 90° and 10 trials at 120° and, for LG, 27 trials at 90° and 34 trials at 120°.

The distance between the proximal and distal ends of each fascicle provided a measure of that fascicle's length throughout the ramped contraction. A two‐dimensional measure of fascicle pennation angle was also obtained from the ultrasound images. This involved fitting a straight line between the ends of the fascicle and a cubic spline to the aponeuroses, and then taking the mean of the included angles between the fascicle and the tangent of the aponeurosis at the two ends of the fascicle. The resulting fascicle length and fascicle pennation angle data were then filtered (zero‐lag, 4th order, 0.5 Hz low‐pass Butterworth).

**Figure 2:**
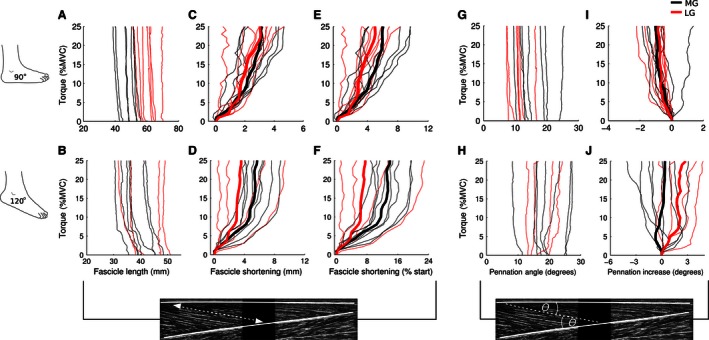
Fascicle length and fascicle pennation angle data from a single subject. (A) With the ankle at 90°, muscle fascicles in the lateral gastrocnemius (LG, red lines) started at a longer length compared to fascicles in the medial gastrocnemius (MG, black lines). All fascicles shortened during the ramped isometric contraction. (C) The magnitude of this shortening varied between 0.2 and 4.5 mm across fascicles; the trimmed means for MG and LG fascicles are presented as bold lines. (E) When expressed as a percentage of the initial length of each fascicle, this corresponded to a shortening of 0.5–9.5%; trimmed means are also presented. MG and LG fascicles shortened to a greater extent when the ankle was at 120° (B, D, F; note the difference in *x*‐axis range). On completion of the ramped contractions, fascicle shortening was greater in MG compared to LG in this subject. (G) With the ankle at 90°, LG fascicle pennation angles were generally smaller than those of MG fascicles. (I) All but one fascicle had a decrease in pennation angle over the course of the ramped contractions. On average, pennation angle decreased 0.8–1.0°. With the ankle at 120° (H, J), LG fascicle pennation angle increased ~2.5° whereas, on average, there was no change in MG fascicles.

The torque signal from each trial was filtered (zero‐lag, 4th order, 0.5 Hz low‐pass Butterworth) and down sampled to 10 Hz. As we were interested in muscle fascicle behavior with respect to changes in torque, all fascicle length and fascicle pennation angle data were linearly interpolated to a common torque‐scale that ranged from 0% to 25% MVC in 0.1% MVC increments, and subsequently averaged over 1% MVC bins. As measured fascicle length and pennation angle vary within and between muscles (e.g., Fig. 2A and G) as well as between subjects, absolute values were computed by subtracting the initial length and pennation angle of each fascicle (i.e., value at 0% MVC) from all other data points (e.g., Fig. 2C and I). Similarly, relative values were computed by expressing changes in fascicle length as a percentage of initial values (e.g., Fig. 2E and F).

In the primary analysis, average MG and LG muscle fascicle behavior was determined for each subject. To improve the precision of muscle architecture estimates (Bolsterlee et al. 2015; Herbert et al. 2015) and reduce the influence of extreme values, a 50% trimmed mean was calculated across all MG and LG fascicles for ramped isometric contractions performed with the ankle at 90° and 120° (e.g., bold lines Fig. 2). Trimmed means were only calculated if four or more fascicles were available for averaging.

In the secondary analysis, average fascicle behavior was determined for the proximal and distal region of the MG muscle for each subject. Proximal fascicles were those recorded from the upper two sites, whereas distal fascicles were those recorded from the lower two sites (see Fig. 1A). As there were only two recording sites for the proximal MG and three sites for the distal MG, all available fascicles from these sites were averaged for ramped contractions performed with the ankle at 90° and 120°.

All analyses were done using Matlab (Mathworks, Natick, MA).

### Statistical analysis

The (fixed) effects of muscle (LG or MG) and angle (90° or 120°) were estimated using linear mixed models. The dependent variables were the slopes of the fascicle length‐torque curve, the normalized fascicle length‐torque curve, and the pennation‐torque curve. The model included an interaction term and had random intercepts for subjects. For all analyses, the interactions terms were small and nonsignificant, so a single estimate of each of the marginal effects of angle and muscle was obtained at the covariate means. All statistical analyses were performed using Stata (StataCorp LP, College Station, TX).

## Results

Mean MG and LG fascicle lengths and pennation angles were obtained for all 15 subjects in ramped isometric contractions performed with the ankle at 90°. More trials were rejected due to poor ultrasound image quality for contractions performed with the ankle at 120° (i.e., with the muscle at shorter lengths); mean fascicle behavior was therefore based on data obtained from 14 subjects for the MG muscle and 10 subjects for the LG muscle. The number of fascicles that contributed to each subject's average are provided in Table 1. Mean MG and LG fascicle lengths and pennation angles at the start of ramped contractions at both ankle angles are provided in Table 2.

**Table 1:** Ankle plantar flexion maximal voluntary contraction (MVC), number of fascicles used to calculate trimmed means for medial gastrocnemius (MG) and lateral gastrocnemius (LG) muscle fascicle behavior for each subject, and total number of subjects included in the final analysis. Also provided are the number of fascicles used to calculate mean muscle fascicle behavior for proximal (MGprox) and distal (MGdist) regions of MG

Ankle angle	MVC (Nm)^a^	Muscle	Fascicle (*n*)^b^	Subjects (*n*)
90°	63 (19.9)	MG	8 [5, 8]	15
		MGprox	2 [1, 2]	15
		MGdist	3 [2, 3]	15
		LG	8 [6, 8]	15
120°	27.4 (9.6)	MG	6 [5, 8]	14
		MGprox	2 [1, 2]	13
		MGdist	3 [1, 3]	14
		LG	7.5 [5, 8]	10

a Values are mean (SD).

b Values are median [range].

**Table 2:** Initial pennation angle and length of medial gastrocnemius (MG) and lateral gastrocnemius (LG) muscle fascicles

Ankle angle	Muscle	Pennation (°)	Length (mm)
90°	MG	16.6 (2.3)	52.2 (8.2)
	LG	11.2 (1.5)	63.2 (10.7)
120°	MG	23.4 (2.6)	38.3 (6.5)
	LG	14.9 (2.7)	47.7 (7.5)

Values are mean (SD).

**Figure 3:**
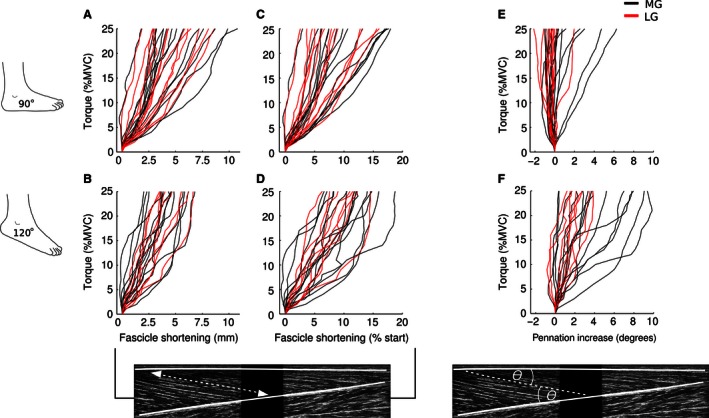
Subject mean fascicle length and fascicle pennation angle. Lines correspond to each subject's trimmed mean. With the ankle at 90° (A, C), medial gastrocnemius (MG) and lateral gastrocnemius (LG) muscle fascicles shortened in all subjects by between 2 and 10 mm (2.5–18%). Overall, the pattern of muscle fascicle shortening was similar when the ankle was at 120° (B, D). Differences between the two ankle positions and the MG and LG were present in pennation angle measures. When the ankle was at 90° (E), mean LG pennation angle decreased slightly in the majority of subjects (0–2°); the majority of MG fascicles experienced little change, although pennation angle did increase 2–6° in a few subjects. When the ankle was at 120° (F), mean pennation angle increased in all subjects: 1–3° in LG fascicles and up to 5–9° in the MG fascicles of several subjects.

**Figure 4:**
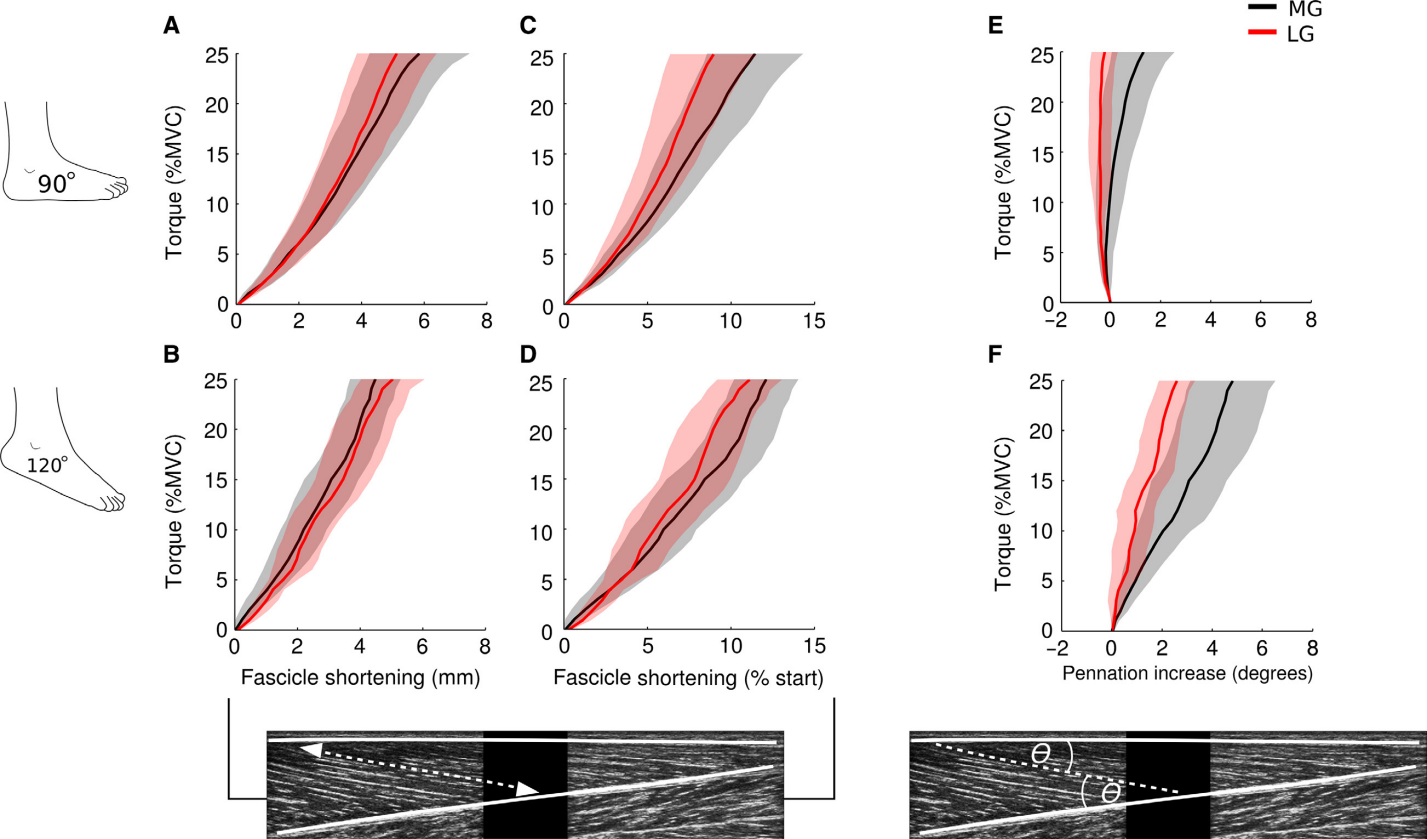
Mean [95% CI] fascicle length and fascicle pennation angle. Changes in muscle fascicle length and pennation angle in the medial gastrocnemius (MG) and lateral gastrocnemius (LG) when the ankle was at 90° (A, C, E) and 120° (B, D, F). Overall, MG and LG fascicles shortened throughout the ramped isometric contractions. Estimates were more variable when the ankle was at 120° because a greater number of trials were rejected due to poor ultrasound image quality. When the ankle was at 90°, the pennation angle of LG muscle fascicles decreased slightly, whereas the pennation angle of MG fascicles increased slightly. When the ankle was at 120°, the pennation angle in MG and LG fascicles increased throughout the ramped contractions.

Changes in fascicle length and pennation angle during ramped contractions are shown for a typical subject in Figure 2. Changes in MG and LG fascicle length and pennation angle averaged across subjects are shown in Figures 3 and 4. In all subjects, fascicles progressively shortened with increased plantar flexion torque. Over the course of the 0–25% MVC contractions, fascicle length decreased between 2 and 10 mm when the ankle was at 90° and between 2 and 7 mm when the ankle was at 120° (Fig. 3A and B). By the end of the ramped contraction, MG muscle fascicles had, on average, shortened by 5.8 mm [4.3–7.3] (mean [95% CI]; 11.1% [7.9–14.3]) when the ankle was at 90° and 4.5 mm [3.6–5.4] (12.1% [10.3–13.9]) when the ankle was at 120° (Fig. 4A–D). Comparatively, LG muscle fascicles shortened by 5.1 mm [3.8–6.4] (8.8% [6.5–11.1]) when the ankle was at 90° and 5.1 mm [4.1–6.1] (11.2% [9.3–13.1]) when the ankle was at 120°. The mixed‐linear models suggest that absolute (mm) and relative (% initial) muscle fascicle length decreased at similar rates when isometric contractions were performed at 90° and 120° (difference in linear slope of −0.026 mm/%MVC [−0.063 to 0.01]; 0.06% initial/%MVC [−0.01 to 0.12]), and that absolute fascicle length decreased at similar rates for MG and LG (−0.002 mm/%MVC [−0.037 to 0.034]; Fig. 4C and D). However, there was a small effect of muscle for relative muscle fascicle length; for every 10% MVC, MG muscle fascicles shortened an additional 0.7% [0.1–1.3] compared to LG fascicles.

In the majority of subjects, there was little change in fascicle pennation angle during ramped contractions when the ankle was at 90° (Fig. 3E), but pennation angle increased by 0.5 to 9° when the ankle was at 120° (Fig. 3F). When the ankle was at 90°, the pennation angle of LG muscle fascicles decreased by 0.3° [−0.2 to 0.8], whereas it is increased by 1.4° [0.2–2.6] in MG fascicles (Fig. 4E). However, when the ankle was at 120°, pennation angle increased linearly during ramped contractions by an average of 2.6° [1.8–3.4] in LG and 4.9° [3.1–6.7] in MG (Fig. 4F).

The mixed‐linear models revealed that ankle angle (90° vs. 120°) and muscle (MG vs. LG) both had effects on the pennation angle of muscle fascicles (Fig. 4E and F). For every 10% MVC, pennation angle increased an additional 1.3° [0.9–1.7] when the ankle was at 120° compared to when it was at 90°. Over the same torque range, pennation angle increased 0.6° [0.2–1.0] more in MG compared to LG.

**Figure 5:**
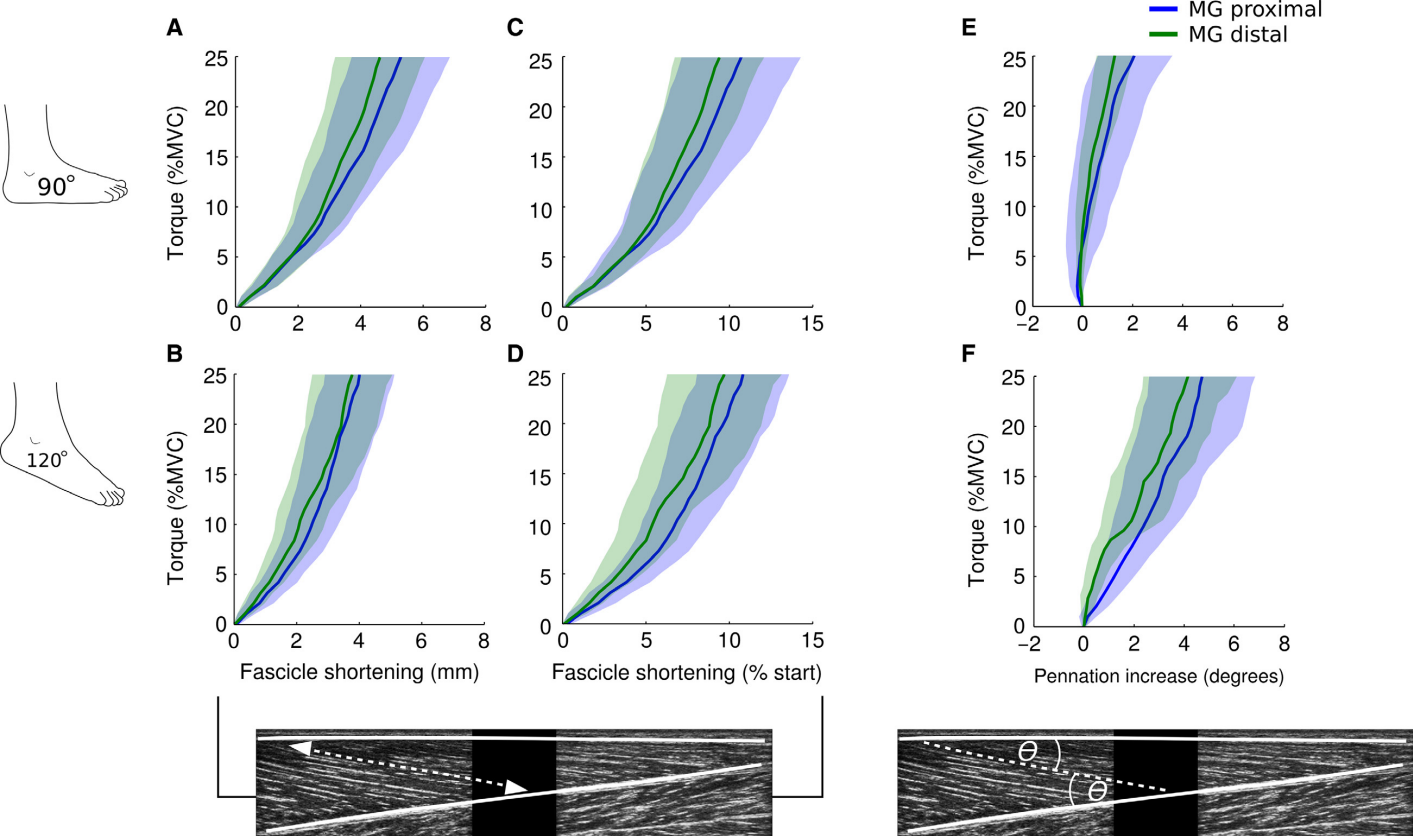
Mean [95% CI] fascicle length and fascicle pennation angle in the proximal and distal regions of the medial gastrocnemius. Changes in muscle fascicle length and pennation angle in the proximal and distal regions of the medial gastrocnemius (MG) when the ankle was at 90° (A, C, E) and 120° (B, D, F). Estimates are more variable due to the inclusion of fewer fascicles in each subject's average and the use of means rather than trimmed means. On average, there was little difference in fascicle shortening between proximally and distally sampled fascicles with the ankle at 90° and 120°. Similarly, there was little difference in fascicle pennation angle.

Changes in fascicle length and pennation angle in the proximal and distal regions of the MG muscle are presented in Figure 5. The overall pattern and magnitude of fascicle shortening was similar between proximal and distal muscle regions at both ankle angles. Likewise, changes in pennation angle were similar between proximal and distal muscle fascicles at 90° and 120°.

## Discussion

This study investigated the continuous change in human gastrocnemius muscle fascicle length and pennation angle during submaximal isometric contractions. In addition to providing precise estimates of mean fascicle behavior during ramped contractions at two ankle angles, these data also show that changes in muscle architecture are similar in proximal and distal muscle regions.

When muscles contract isometrically, fascicles shorten as tendons and aponeuroses lengthen (Kawakami et al. 2000; Finni 2006). In this study, gastrocnemius muscle fascicles shortened during contractions to 25% MVC by, on average, 4–6 mm, which corresponds to 8–12% of their initial length. Similarly, Narici et al. (1996) previously reported 4–5 mm (8–10%) shortening in MG muscle fascicles when brief 20–30% MVC contractions were performed with the ankle at 110°. On the other hand, Maganaris et al. (1998) reported fascicle shortening of ~5 mm (7%) in MG and ∼13 mm (18%) in LG during brief 20–30% MVC isometric contractions with the ankle at 90°. Compared to our results, the average LG fascicle shortening reported by Maganaris et al. (1998) corresponds to the upper limit of that observed in any of our subjects (see Fig. 3). However, the initial length of LG fascicles in our study was ~10 mm shorter and, as previously shown by Arampatzis et al. (2006) for maximal contractions, shorter fascicle lengths are associated with less fascicle shortening during isometric contractions. Furthermore, the ultrasound transducer used by Maganaris et al. (1998) had a small field of view that forced them to extrapolate the intersection between the aponeuroses and fascicles. Both these explanations could account for this difference in observed fascicle shortening.

In pennate muscles, pennation angle typically increases with increases in isometric torque (Herbert and Gandevia 1995; Narici et al. 1996; Maganaris et al. 1998). In line with this, fascicle pennation angle increased almost linearly during ramped contractions with the ankle at 120° (see Fig. 4). A similar 4° increase in MG pennation angle was observed by Narici et al. (1996) during contractions of similar intensity with the ankle at 110°. Contrary to the proposed relationship between pennation angle and isometric torque, we found that mean fascicle pennation angle decreased ~0.5° in LG and increased ~1° in MG with the ankle at 90°; this effect of joint angle on change in pennation was confirmed by the mixed‐linear model. These values are somewhat smaller than the 4–5° increase in pennation angle reported by Maganaris et al. (1998) at the same joint angle using their interpolated measures of pennation angle. Overall, when the ankle is at 90°, it appears there is little change in gastrocnemius fascicle pennation during low‐to‐moderate isometric contractions. There was also a small difference in the rate at which pennation angle increased depending on whether fascicles were located in MG and LG. This effect was an order of magnitude smaller than the effect of joint angle: 1.5° over the range of torques investigated (i.e., 0–25% MVC).

Muscle fascicles located in proximal and distal regions of MG shortened similarly over the course of ramped isometric contractions. Importantly, fascicle shortening started immediately at contraction onset in both muscle regions. There was no evidence to support previous reports that shortening of the distal portion of the gastrocnemius muscle starts earlier due to a higher distal concentration of low‐threshold motor units (Vieira et al. 2011; Hodson‐Tole et al. 2013). Care must be taken when interpreting our results because few trials were averaged at each site. Thus, these preliminary findings should be confirmed in a larger sample of subjects, averaging across a greater number of trials. Regional muscle activation and hypertrophy have been reported in the human vastus lateralis muscle, a pennate muscle that inserts on a much shorter, and likely less compliant tendon (Franchi et al. 2014; Gallina et al. 2016). It would be useful to determine how fascicle length and pennation angle changes in ramped isometric contractions in pennate muscles with with shorter, stiffer tendons.

Studies investigating changes in gastrocnemius muscle architecture during static contractions often have small sample sizes (6 or 7 subjects; Narici et al. 1996; Kawakami et al. 1998; Maganaris et al. 1998; Muramatsu et al. 2002a,b; Namburete and Wakeling 2012). In addition, results from these studies often reflect values extracted from single trials or the average of 2–3 trials. This practice is problematic due to the limitations of ultrasound‐based recordings. Comparing MG architectural parameters measured at rest with ultrasound and MRI‐based diffusion tensor images, Bolsterlee et al. (2015) demonstrated that ultrasound measures from single recordings generate imprecise estimates, with a mean absolute difference between ultrasound and MRI‐based measurements of 10 mm. Although diffusion tensor imaging has yet to be used in active conditions for this muscle, there is evidence that ultrasound measures are likely to be even less precise under active conditions (Maganaris et al. 1998). To address this limitation, Bolsterlee et al. (2015) and Herbert et al. (2015) recommend averaging several ultrasound measurements to improve precision. In this study we measured muscle fascicle behavior from a total of 14 sites, eight over MG and six over LG, to improve the precision of our measures. Furthermore, our sample size was 15, which provides a better estimate of overall mean fascicle behavior and a better estimate of the normal range of values that can be expected across subjects than that provided by earlier studies with six or seven subjects.

This study has some limitations. It was recently shown that, in muscles at rest, pressure from the ultrasound probe on the skin surface causes measures of pennation angle taken with respect to the superficial aponeurosis to be underestimated by 10° (Bolsterlee et al. 2015). This artifact has yet to be investigated in active muscles, but may be less problematic under active conditions. Because of this, and the fact that previous studies have quantified pennation as the average of the included angles between fascicle and both aponeuroses, we opted to use the traditional measure of pennation angle. Nevertheless, it is possible that our measures are slightly biased toward smaller pennation angles. Another issue is that our data are limited to 25% of the torque generating capacity of the plantar flexor muscles. While this torque range is the most important in terms of daily activities (Kern et al. 2001), some muscle models require maximal values to be provided; our results are of limited use to such applications. While it would have been possible to analyse select trials from a few subjects at higher contraction intensities, the few resulting estimates would have been imprecise. As a final limitation, LG data recorded with the ankle at 120° were not included for five subjects due to a large number of trials that had to be rejected due to poor image quality. Nonetheless, LG data from the remaining 10 subjects were quite consistent and thus likely provide a reasonable estimate.

This study is the first to describe the continuous change in fascicle length and pennation angle in the human gastrocnemius muscle during ramped isometric contractions and that these changes in muscle architecture are similar in proximal and distal muscle regions. It is hoped that these results will prove useful to future studies modeling active force generation and changes in muscle mechanics.

## Conflict of Interest

None declared.
